# Hope and Distress Symptoms of Oncology Patients in a Palliative Care Setting

**DOI:** 10.7759/cureus.38041

**Published:** 2023-04-24

**Authors:** Maria Nikoloudi, Eleni Tsilika, Sotiria Kostopoulou, Kyriaki Mystakidou

**Affiliations:** 1 Pain Relief and Palliative Care Unit, Department of Radiology, Areteion Hospital, School of Medicine, National and Kapodistrian University of Athens, Athens, GRC

**Keywords:** palliative care, cancer, depression, anxiety, hope

## Abstract

Background

Hope has a positive impact on health, playing a significant role in managing illness and its associated losses. In oncology patients, hope is crucial for effective adaptation to the disease, as well as a strategy for coping with physical and mental distress. It enhances disease management, psychological adaptation, and overall quality of life. However, due to the complexity of the effect of hope on patients, particularly those under palliative care, identifying its relationship with anxiety and depression remains a challenge.

Methodology

In this study, 130 cancer patients completed the Greek version of the Herth Hope Index (HHI-G) and the Hospital Anxiety and Depression Scale (HADS-GR).

Results

The HHI-G hope total score was strongly negatively correlated with HADS-anxiety (r = -0.491, p < 0.001) and HADS-depression (r = -0.626, p < 0.001). Patients with performance status, as defined by the Eastern Cooperative Oncology Group (ECOG), of 0-1 without radiotherapy had higher HHI-G hope total scores compared to those with ECOG status 2-3 (p = 0.002) and radiotherapy (p = 0.009). Multivariate regression analysis showed that patients who received radiotherapy had 2.49 points higher HHI-G hope scores compared to those who did not (explaining 3.6% of hope). An increase of 1 point in depression led to a 0.65-point decrease in the HHI-G hope score (explaining 40% of hope).

Conclusions

A deeper understanding of common psychological concerns and hope in patients with serious illnesses can improve their clinical care. Mental health care should focus on managing depression and anxiety, as well as other psychological symptoms, to enhance and maintain patients’ hope.

## Introduction

Living with cancer poses numerous challenges that can impact a person both psychologically and behaviorally. The patient must learn to cope with the loss of their health and functionality and create a new narrative for their changed life. In recent times, hope has gained importance in clinical settings due to its therapeutic potential [1]. Hope has evolved from being a unidimensional motivational force to a multidimensional one [2]. It is defined as a positive motivational and cognitive attribute that is necessary to initiate and sustain action toward goal attainment [3,4].

Hope has been linked to happiness, achievement, and health [5]. It acts as a protective mechanism against stress and disease, enhances psychological adaptation and quality of life, and is a prerequisite for effective response to treatment. A relationship between hope, self-esteem, and life satisfaction has also been noted [6,7].

Studies have shown that hope is associated with the presence of significant others, personal health, and functionality. It is viewed as a life force that gives strength to live long enough to participate in family events. Healthcare providers view hope in terms of future orientation, pain management, meeting the patient’s needs, and a wish for a peaceful death [8].

The most widely used tools to measure hope are the Adult Hope Scale, the Herth Hope Scale (HHS), and its short form, the Herth Hope Index (HHI) [7]. The HHI has been developed to assess hope in clinical settings with ill and elderly populations and has been translated and validated in various cultures [1,9].

However, some studies have found different factor solutions for the HHI due to translation and cultural differences in the understanding of hope [9-11].

This study aims to further the growing literature on hope by assessing the relationships between hope, depression, and anxiety in palliative care cancer patients using the Greek version of the Herth Hope Index (HHI-G) and the Hospital Anxiety and Depression Scale (HADS-GR) [11,12].

## Materials and methods

This correlation study was conducted at the outpatient clinic of the Pain Relief and Palliative Care Unit in Athens, Greece. A total of 130 eligible cancer patients referred to the unit for pain relief and management of symptoms were included in this study. Data were collected from August 2018 to April 2019. The inclusion criteria were histologically confirmed malignancy, age 18 years or older, the ability to communicate effectively, and provision of informed consent. Exclusion criteria included a history of drug abuse, unawareness of their disease diagnosis, and significant cognitive impairment (Montreal Cognitive Assessment ≤26, Mini‐Mental State Examination ≤24) [13].

A member of the palliative care unit conducted brief interviews with each patient to collect background medical history, demographic data, prior mental health status, and current condition. Patients were approached on a face-to-face basis and were informed of the nature of the study, as well as the risks and benefits of participation. Participants who consented provided written informed consent.

Measurements

The study utilized a self-report questionnaire package that included the below measures.

Identification Questionnaire

Developed by the authors, it consisted of demographic information, disease status, treatment regimen (surgery, chemotherapy, radiotherapy, and opioids), and performance status, as defined by ECOG. Patients with an ECOG score of 0 or 1 were classified as having a good performance status, while those with a score of 2 or 3 were considered to have a moderate to poor performance status [14].

HHI-G

The HHI is a 12-item adapted version of the HHS that has been translated and validated in various cultures [9,15]. The HHI measures hope, with scores ranging from 12 to 48 points, with higher scores indicating a higher level of hope. The HHI-G was translated, validated, and evaluated for its psychometric properties in palliative care cancer patients by Nikoloudi et al. [11]. In this study, the authors adopted a single-factor model, in line with previous studies conducted in Italy and Germany [9,16].

HADS-GR

The HADS is a self-assessment scale specifically designed for use in hospital departments [17]. It provides clinically meaningful results as a psychological screening tool, in clinical group comparisons, and in studies assessing several aspects of disease and quality of life. The two subscales, HADS-anxiety and HADS-depression, each consist of seven items, each rated on a four-point scale (0 = no problems, 3 = maximum distress) [17].

In the original study, the authors recommended three cut-off scores for both subscales, namely, 0-7 for non-cases, 8-10 for doubtful cases of anxiety and depression (possible scores range from 0-21 for each subscale), and ≥11 for cases. Many investigators have interpreted the HADS as a bi-dimensional instrument that independently assesses anxiety and depression [18]. The HADS scale has been standardized in a sample of advanced Greek cancer patients, proving to be a useful screening measure for anxiety and depression in this patient population [12].

Ethical considerations

The study protocol was approved by the Areteion Hospital Ethics Committee (approval number: 113/13-02-2019) and was conducted in accordance with Good Clinical Practice guidelines and the Declaration of Helsinki. All participants were informed about the study’s objectives and provided written informed consent.

Statistical analysis

Data were expressed as mean ± SD for quantitative variables and as percentages for qualitative variables. The Kolmogorov-Smirnov test was used for the normality analysis of quantitative variables. Unifactorial analysis was performed using the Student’s t-test, one-way analysis of variance, and Pearson and Spearman correlation coefficients to analyze the relationship between the outcome variable (HHI total score) and demographic and clinical characteristics. All demographic and clinical variables with a p-value <0.2 in bivariate analyses with HADS subscales were included in a multiple linear regression model using the enter method to identify the most significant independent factors associated with the outcome variable. All assumptions of linear regression analysis (homoscedasticity, linearity, normality, independence of error terms, and multicollinearity of independent variables) were examined. All tests were two-sided, and statistical significance was set at p-values <0.05. All analyses were performed using SPSS version 21.00 (IBM Corp., Armonk, NY, USA).

## Results

Descriptive results

The average age of the patients was 67 years (range = 29-90 years), with 47% of the participants being male and 53% female. Overall, 21% of the participants had a primary school education, 55.4% had a secondary school education, and 24% had a university education. Furthermore, 65.4% of the patients were married, 8.5% were single, 20.8% were divorced, and 54% were widows. Regarding ECOG status, 57.7% of the patients had a good performance status (ECOG 0-1), while 42.3% had a moderate-to-poor performance status (ECOG 2-3). There were no patients with a score of 4. Overall, 64.6% of the patients had undergone chemotherapy, 68.5% had received radiotherapy, and 59.2% had undergone surgery (Table 1).

**Table 1:** Demographic and clinical characteristics. N: frequency; %: the percentage; ECOG: Eastern Cooperative Oncology Group

Factor	Categories	Ν	%
Age (mean ± SD)	67.00±12.03		
Gender	Male	61	46.9
	Female	69	53.1
Family status	Married	85	65.4
	Unmarried	11	8.5
	Divorced	27	20.8
	Widow	7	5.4
Education	Primary	27	20.8
	Secondary	72	55.4
	University	31	23.8
ECOG	0-1	75	57.7
	2-3	55	42.3
Chemotherapy	No	46	35.4
	Yes	84	64.6
Radiotherapy	No	41	31.5
	Yes	89	68.5
Surgery	No	53	40.8
	Yes	77	59.2

The results of the internal consistency of hope (HHI-G), HADS-anxiety, and HADS-depression scores exceeded the acceptable limits of internal coherence (α ≥ 0.7) with α = 0.860, α = 0.892, and α = 0.848, respectively. The average HHI-G total score was 36.68 ± 6.05, indicating a high level of hope in the sample. The mean HADS-anxiety score was 7.44 ± 4.31, indicating low anxiety in the sample, while the mean HADS-depression score was 6.41 ± 5.08, indicating low depression.

There were no statistically significant differences between the two sexes (p = 0.448), among marital status groups (p = 0.488), among educational levels (p = 0.250), between those who received chemotherapy and those who did not (p = 0.940), and between those who underwent surgery and those who did not (p = 0.103). However, there was a statistically significant difference between those with ECOG status 2-3 and those with ECOG status 0-1 (p = 0.002). There was also a significant difference between those who received radiotherapy and those who did not with regard to hope (p = 0.009). There was no statistically significant correlation between hope and age (p = -0.098), but there was a correlation between hope and anxiety (p < 0.001) and depression (p < 0.001) (Table 2).

**Table 2:** Unifactorial analysis of HHI total score. HHI: Herth Hope Index; ECOG: Eastern Cooperative Oncology Group; HADS: Hospital Anxiety and Depression Scale

Factor	Categories	Mean ± SD	P-value
Gender	Male	37.11 ± 5.86	0.448
	Female	36.30 ± 6.24	
Family status	Married	37.26 ± 5.86	0.488
	Unmarried	35.91 ± 4.89	
	Divorced	35.74 ± 7.43	
	Widow	34.57 ± 3.51	
Education	Primary	35.19 ± 5.99	0.250
	Secondary	37.40 ± 6.23	
	University	36.32 ± 5.57	
ECOG	0-1	38.09 ± 5.73	0.002
	2-3	34.76 ± 5.99	
Chemotherapy	No	36.63 ± 5.39	0.940
	Yes	36.71 ± 6.41	
Radiotherapy	No	34.66 ± 5.17	0.009
	Yes	37.62 ± 6.23	
Surgery	No	35.64 ± 5.88	0.103
	Yes	37.40 ± 6.10	
Age	Pearson’s r = -0.098		0.266
HADS-anxiety	Spearman’s r = -0.491		<0.001
HADS-depression	Spearman’s r = -0.626		<0.001

Unifactorial analysis of HHI-G total score

The unifactorial analysis showed that the HHI-G hope total score was highly negatively correlated with HADS-anxiety (r = -0.491, p < 0.001) and HADS-depression (r = -0.626, p < 0.001). Patients with ECOG status 0-1 who did not receive radiotherapy had higher values of HHI-G hope total score compared to those with ECOG status 2-3 (p = 0.002) and those who received radiotherapy (p = 0.009). There were no statistically significant associations between the HHI-G hope total score and other factors (Table 2).

Multifactorial analysis of HHI-G total score

A stepwise multiple regression model was used to examine the contribution of demographic, clinical, and HADS-anxiety and depression variables to the participants’ level of HHI-G hope. The results of the regression analysis showed decreased values of HADS-depression (beta coefficient ± SE = -0.645 ± 0.127; R^2^ = 40%; p < 0.001) and patients’ radiotherapy (beta coefficient ± SE = 2.49 ± 0.91; R^2^ = 3.6%; p = 0.007) (Table 3).

**Table 3:** Multifactorial analysis of HHI total score. HHI: Herth Hope Index; ECOG: Eastern Cooperative Oncology Group; HADS: Hospital Anxiety and Depression Scale

Factor	Reference category	R^2^	Beta coefficient	SE	P-value
Age	---	0.7%	-0.047	0.035	0.185
ECOG	0-1	0.1%	0.453	0.970	0.641
Radiotherapy	No	3.6%	2.489	0.910	0.007
Surgery	No	0.5%	-0.871	0.882	0.325
HADS-anxiety	---	1.2%	-0.227	0.135	0.095
HADS-depression	---	40%	-0.645	0.127	<0.001

## Discussion

In our study, we examined the relationship between hope and psychological distress (depression and anxiety) in cancer patients and evaluated the impact of their clinical and demographic characteristics on hope. The findings indicate that higher levels of hope are associated with lower levels of depression and anxiety symptoms, as previously reported in the literature [19]. Our analysis showed that depression and anxiety were inversely related to hope, with high negative correlations. Patients with ECOG performance status 0-1 who did not undergo radiotherapy had higher hope scores, while patients with ECOG performance status 2-3 who underwent radiotherapy had lower hope scores. The results showed that performance status has a significant impact on anxiety and depression symptoms, with patients with poor performance status tending to have higher levels of these symptoms. This finding is consistent with previous studies [20]. The study results align with those of Li et al. [21], who found that cervical cancer patients undergoing radiotherapy maintained moderate-to-high levels of hope, despite the physical and mental stress they experienced. This is in line with the study by Yanling et al. [22] which reported a similar pattern. The HHI-G was used to measure hope levels, with a higher score indicating a higher level of hope, as there are no established cut-off scores for the scale. The mean score of the HHI-G hope total score was 36.68 ± 6.05, indicating high levels of hope in the study sample. This is similar to previous studies, such as Wahl et al. [23] (mean score 36.7 ± 4.2) in healthy adults and Nekolaichuk and Bruera [24] (mean score 37 ± 5) in advanced cancer patients. Benzein and Berg [25] found a mean HHI score of 39.6 in patients receiving palliative care in Sweden. The mean score of HADS-anxiety was 7.44 ± 4.31, indicating low levels of anxiety, and the mean score of HADS-depression was 6.41 ± 5.08, indicating low levels of depression, which agrees with the findings of Carroll et al. [18].

The results of this study showed no significant difference in hope levels between male and female patients or between married and unmarried patients, which aligns with the findings of Bjornnes et al. [26]. Previous research has indicated that hope may be impacted by factors such as older age, lower education, and lower socioeconomic status. Cancer and its related challenges can lead to feelings of despair and loss of hope, but hopeful thinking can help patients better cope with the diagnosis and manage their symptoms. While some patients may experience less distress, others may struggle with hopelessness during the final stages of their illness. Despite its importance, addressing hope, depression, and anxiety in palliative care for cancer patients is often neglected in clinical practice, but can be a critical aspect of psychological well-being [27]. Studies have shown that various patient characteristics such as age, gender, and marital status can impact hope levels in cancer patients [28]. Additionally, depression has been found to negatively impact hope in cancer patients [16]. The literature suggests that hope can be both preventative and therapeutic for individuals facing stressors such as a serious illness. Patients who adopt a hopeful mindset tend to better handle such situations by breaking down their goals into smaller, achievable steps, leading to less distress and more effective adaptation to the situation.

A study by Ahari et al. [29] further supported this by showing that hope therapy increased hope and reduced depression in mothers of children diagnosed with cancer. This highlights the importance of promoting hope-boosting interventions for cancer patients in palliative care to address their specific needs.

Limitations

This study has the limitations commonly encountered in studies using cross-sectional data, which do not permit conclusions about the direction of the relationship between hope and depression and anxiety. Therefore, it is difficult to establish causality. Additionally, we did not assess the intensity or persistence of pain, which is an important aspect to consider when investigating hope in illness. Bjornnes et al. have shown that higher levels of pain intensity are associated with lower levels of hope [26].

## Conclusions

The study aimed to examine the relationship between hope, depression, and anxiety among patients in a Greek palliative care facility. The results support the idea that psychological support for cancer patients should focus on providing clear and immediate goals that promote hope and reduce depression.

Hope is crucial for coping with cancer as it facilitates acceptance of the illness, improves coping capacity, and encourages positive behaviors related to treatment. Treatment can exacerbate anxiety and hopelessness due to its impact on the immune system. Assessing hope enables the implementation of interventions that can benefit patients with chronic diseases and their families.

A deeper understanding of the psychological challenges and hope experienced by patients with serious illnesses can enhance the quality of their clinical care. Mental health care for terminal illnesses should prioritize managing uncontrolled psychological symptoms such as unrecognized and untreated depression and anxiety and their relationship with hope. The findings of this study can provide valuable information and motivate healthcare professionals to understand the significance of hope and its impact on depression and anxiety. It is important to highlight the need to detect the influence of hope on patients’ depression and anxiety to develop effective treatment strategies.
